# MALAT1-regulated gene expression profiling in lung cancer cell lines

**DOI:** 10.1186/s12885-023-11347-7

**Published:** 2023-09-04

**Authors:** Jungwook Roh, Boseong Kim, Mijung Im, Wonyi Jang, Yeonsoo Chae, JiHoon Kang, BuHyun Youn, Wanyeon Kim

**Affiliations:** 1 Department of Science Education, Korea National University of Education, Cheongju-si, 28173 Chungbuk Republic of Korea; 2 Department of Hematology and Medical Oncology, Winship Cancer Institute of Emory, Emory University School of Medicine, Atlanta, GA 30322 USA; 3 Department of Biological Sciences, Pusan National University, Busan, 46241 Republic of Korea; 4 Department of Biology Education, Korea National University of Education, Cheongju-si, 28173 Chungbuk Republic of Korea

**Keywords:** MALAT1, LncRNA, NSCLC, Lung Cancer, Bioinformatics

## Abstract

**Background:**

Non-small cell lung cancer (NSCLC) is the most common type of lung cancer and has a poor prognosis. Identifying biomarkers based on molecular mechanisms is critical for early diagnosis, timely treatment, and improved prognosis of lung cancer. MALAT1 has been reported to have overexpressed and tumor-promoting functions in NSCLC. It has been proposed as a potential biomarker for the diagnosis and prognosis of cancer. Therefore, this study was conducted to profile the changes in gene expression according to the regulation of expression of MALAT1 in NSCLC cell lines and to investigate the correlation through bioinformatic analysis of differentially expressed genes (DEGs).

**Methods:**

MALAT1 expression levels were measured using RT-qPCR. The biological functions of MALAT1 in NSCLC were analyzed by cell counting, colony forming, wound-healing, and Transwell invasion assays. In addition, gene expression profiling in response to the knockdown of MALAT1 was analyzed by transcriptome sequencing, and differentially expressed genes regulated by MALAT1 were performed by GO and KEGG pathway enrichment analyses. Bioinformatic databases were used for gene expression analysis and overall survival analysis.

**Results:**

Comparative analysis versus MALAT1 expression in MRC5 cells (a normal lung cell line) and the three NSCLC cell lines showed that MALAT1 expression was significantly higher in the NSCLC cells. MALAT1 knockdown decreased cell survival, proliferation, migration, and invasion in all three NSCLC cell lines. RNA-seq analysis of DEGs in NSCLC cells showed 198 DEGs were upregulated and 266 DEGs downregulated by MALAT1 knockdown in all three NSCLC cell lines. Survival analysis on these common DEGs performed using the OncoLnc database resulted in the selection of five DEGs, phosphoglycerate mutase 1 (*PGAM1*), phosphoglycerate mutase 4 (*PGAM4*), nucleolar protein 6 (*NOL6*), nucleosome assembly protein 1 like 5 (*NAP1L5*), and sestrin1 (*SESN1*). The gene expression levels of these selected DEGs were proved to gene expression analysis using the TNMplot database.

**Conclusion:**

MALAT1 might function as an oncogene that enhances NSCLC cell survival, proliferation, colony formation, and invasion. RNA-seq and bioinformatic analyses resulted in the selection of five DEGs, *PGAM1*, *PGAM4*, *NOL6*, *NAP1L5*, and *SESN1*, which were found to be closely related to patient survival and tumorigenesis. We believe that further investigation of these five DEGs will provide valuable information on the oncogenic role of MALAT1 in NSCLC.

**Supplementary Information:**

The online version contains supplementary material available at 10.1186/s12885-023-11347-7.

## Introduction

Lung cancer has high incidence and mortality rates worldwide and is classified as small cell lung cancer or non-small cell lung cancer (NSCLC) according to its histological characteristics [1–3]. NSCLC is the most common type and accounts for 80 ~ 85% of cases [4]. Despite therapeutic advances, the prognosis of NSCLC remains poor [5], and thus it is important that molecular biomarkers related to tumorigenic mechanisms be identified that enable early diagnosis and timely treatment.

Long non-coding RNAs (lncRNAs) are defined as transcripts of more than 200 nucleotides that do not code for proteins and are molecular biomarker candidates [6]. LncRNAs are known to regulate mRNA junctions and act as precursors for other non-coding RNAs, such as microRNAs (miRNAs). Furthermore, lncRNAs have been suggested to have oncogenic or tumor-suppressive roles and are known to participate in diverse signaling pathways in cancer [7, 8]. In addition, they are known to contribute to the occurrence and development of various cancers, including liver, brain, breast, and lung cancer [9–11]. Studies on the roles of lncRNAs have recently been used to investigate the molecular mechanisms involved in malignant processes, such as carcinogenesis and metastasis [12, 13]. Moreover, by analyzing lncRNA expressions in peripheral blood, urine sediment, and tissue samples, various lncRNAs have been shown to be potential additional or independent biomarkers for cancer diagnosis or prognosis [7].

Nuclear lncRNA metastasis-associated lung adenocarcinoma transcript 1 (MALAT1) is a highly preserved lncRNA and abundant in cells and tissues [14]. MALAT1 is located in chromosome 11q13.1, has 8,700 nucleotides, and is an essential member of the lncRNA family [15]. MALAT1 was first discovered in NSCLC and is known to be involved in the regulation of cell cycle and migration [14, 15]. MALAT1 has also been reported to promote tumorigenesis in several cancers. In NSCLC, MALAT1 is overexpressed and interacts with several miRNAs to regulate cell proliferation, metastasis, and apoptosis [16, 17]. MALAT1 functions as an oncogene by acting as a sponge for miR-34a and upregulating cyclin D1 expression in osteosarcoma [18]. In prostate cancer, MALAT1 acts as a competitive endogenous RNA for miR-1 and is associated with cell proliferation, invasion, and apoptosis [19, 20]. MALAT1 also inhibits apoptosis by upregulating the anti-apoptotic members Bcl-2 and Bcl-xL [21]. In addition, MALAT1 could reduce cisplatin sensitivity in NSCLC by regulating the expression of miR-145 and KLF transcription factor 4 [22]. It was found that MALAT1 could suppress the inhibitory effect of polyphyllin I (PPI) and PPI-induced apoptosis on gefitinib-resistant NSCLC cells [23]. Since MALAT1 plays a critical role in tumor development, employing antisense oligonucleotides, small interfering RNA (siRNA), or the CRISPR system might be a promising therapeutic strategy targeting MALAT1 [16, 24]. Besides, MALAT1 is a relatively stable transcript that can be detected in tumor tissue and body fluids with a half-life of 9 to 12 h [25]. Taken together, MALAT1 has potential use as a biomarker for cancer diagnosis and prognosis [16].

Bioinformatic tools have become increasingly important in cancer research as they can be used to identify biological functions and signaling pathways associated with cancer genes. Moreover, these tools have facilitated the identification of potential biomarker candidates. In this study, we profiled differentially expressed genes (DEGs) significantly altered by MALAT1 knockdown in NSCLC cells. Furthermore, DEGs closely related to NSCLC development were selected through bioinformatic analyses, and the relationships between selected DEGs and NSCLC were identified. Our results provide underlying information on the molecular activities of MALAT1.

## Materials and methods

### Cell-preparation

Cell culture and transfection were performed as previously described [26]. A normal lung cell line (MRC5) and three NSCLC cell lines (H460, A549, H1299) were obtained from the American Type Culture Collection (ATCC, Manassas, VA, USA). Cells were cultured in RPMI-1640 (Hyclone, Marlborough, MA, USA) or DMEM (Hyclone) supplemented with 10% FBS (Hyclone), 100 U/ml penicillin, and 100 µg/ml streptomycin (Gibco, Waltham, MA, USA) at 37 ℃ in a 5% CO_2_/95% air atmosphere.

Each cell line was divided into negative controls, and si-Control (Invitrogen, Waltham, MA, USA) treated and si-MALAT1 (Invitrogen) treated cells. Cells (70–80% confluent) from each si-Control group and si-MALAT1 group were transiently transfected with siRNAs (10 µM) for control (si-Control) or MALAT1 knockdown (si-MALAT1) using Lipofectamine RNAiMAX (Invitrogen). For cell viability and motility evaluations, cells were incubated for 24 h post-transfection.

### Total RNA-preparation

After transient transfection, total RNA was isolated using TRIzol (Invitrogen), as previously described [27]. RNA qualities and concentrations were assessed using a Nanodrop 2000 (Thermo Scientific, Waltham, MA, USA) and an OD 260/280 ratio. Extracted RNAs were used for reverse transcription quantitative real-time polymerase chain reaction (RT-qPCR) and transferred to Macrogen Inc. (Macrogen, Seoul, Republic of Korea) for transcriptome sequencing.

### RT-qPCR

First-strand cDNA was generated from total RNA using the M-MLV reverse transcriptase system (Bioneer, Daejeon, Republic of Korea). The conditions used for reverse transcription (RT) were 60 min at 37 ℃ and 5 min at 99 ℃. RT products were stored at 4 ℃ for 30 ~ 40 min, and then subjected to RT-qPCR using specific primers (Supplementary Table 1). The expression levels of RT products were determined using GoTaq® qPCR Master Mix (Promega, Madison, WI, USA) according to the manufacturer’s protocol and normalized versus U6. Relative RNA expression levels were calculated at least three times using the comparative 2^−ΔΔCt^ method.

### Cell viability assays

Cell counting was performed using trypan blue solution (Gibco) [28]. Briefly, cells (1 × 10^5^) were plated on 60 mm plates for 24 h and transfected with 2 µL (10 µM) si-Control or 2 µL (10 µM) si-MALAT1. Cells were separated with 10% trypsin-EDTA (Gibco), washed with PBS, resuspended in PBS, and diluted 1:1 with trypan blue solution. Short-term viabilities were determined by counting the number of surviving cells for 0 ~ 5 days.

Colony formation assays were used to determine long-term cell viabilities. H460 cells were seeded at 500 cells per 35 mm dish, and H1299 and A549 cells were seeded at 400 cells per 35 mm dish [29]. Cells were transfected with 1 µL (10 µM) si-Control or 1 µL (10 µM) si-MALAT1 48 h later and incubated in a 5% CO_2_/95% air atmosphere for 12 days at 37 ℃. Colonies containing 50 or more cells were imaged and scored as the number of colonies. The resulting colonies were washed twice with PBS fixed with 10% methanol/10% acetic acid, and stained with 1% crystal violet (Sigma, St. Louis, MO, USA).

### Cell motility assays

A wound-healing assay was used to investigate the effect of MALAT1 on cell motility [30]. Non-treated cells and cells transient transfected with 2 µL (10 µM) si-Control or 2 µL (10 µM) si-MALAT1 were grown to 80% confluence in 60 mm dishes at 37 ℃ in 5% CO_2_. After 6 h, cells were scraped with 200 µL pipette tip, washed with PBS to remove non-attached cells, and incubated in fresh culture medium for 24 ~ 48 h. Areas covered by migrating cells were then determined using photomicrographs taken at × 100 using an Olympus CKX 53 inverted microscope (Olympus Optical, Tokyo, Japan).

Cell invasion analysis was performed using 24-well Transwell chambers with 8.0 μm pore polycarbonate membranes (Corning, Bedford, MA, USA). Transwell invasion assays were performed as previously described [31]. NSCLC cells (5 × 10^4^) were seeded in membranes pre-coated with Matrigel (Corning) in upper chambers. Then, the cells were transfected with 0.1 µL (10 µM) si-Control or 0.1 µL (10 µM) si-MALAT1. After 24 h incubation, the cells in upper chambers were removed with cotton swabs, and cells that had migrated to lower membrane surfaces were fixed and stained with a 5% crystal violet. Photomicrographs were taken at × 400 using an Olympus CKX53 inverted microscope (Olympus Optical). Three images of 10 random fields per membrane were captured, and the number of cells that migrated to lower membrane surfaces were counted. All experiments were repeated three times.

### Statistical analysis

Statistical analysis was performed using Prism 5 software (GraphPad Software, San Diego, CA, USA), and statistical significance was accepted for p-value < 0.05. All experiments were performed independently in triplicate. The ranking of experimental results was performed by one-way or two-way ANOVA. After analysis by one-way ANOVA, Tukey’s honestly significant difference test was performed, whereas after analysis by two-way ANOVA, Bonferroni’s post-test was performed.

### Transcriptome sequencing

Total RNA concentrations were evaluated using Quant-IT RiboGreen (Invitrogen). In order to assess the integrity of total RNA, samples were run on TapeStation RNA Screen Tapes (Agilent, Santa Clara, CA, USA). Only high-quality RNA preparations with RIN values ​​> 7.0 were used to construct RNA libraries. Libraries were independently prepared using 0.5 µg of total RNA per sample using the Illumina TruSeq Stranded Total RNA Library Prep Gold Kit (Illumina, San Diego, CA, USA). After rRNA had been removed from total RNA using the Ribo-Zero rRNA Removal Kit (Human/Mouse/Rat Gold) (Illumina), the remaining mRNA was fragmented into small pieces using divalent cations at elevated temperatures.

Cleaved RNA fragments were copied into first-strand cDNA using SuperScript II reverse transcriptase (Invitrogen) and random primers. This was followed by second-strand cDNA synthesis using DNA polymerase I, RNase H, and dUTP. cDNA fragments were subjected to the final repair, single ‘A’ base addition, and adapter ligation. Products were then purified and concentrated by PCR to generate the final cDNA library. Libraries were quantified using the KAPA Library Quantification Kit for Illumina Sequencing Platforms according to the qPCR Quantification Protocol Guide (Kapa Biosystems, Wilmington, MA, USA) and then validated using a TapeStation D1000 ScreenTape (Agilent). Indexed libraries were then submitted to Illumina NovaSeq (Illumina), and paired-end (2100 bp) sequencing was performed at Macrogen Inc. [32]. The sequencing results were deposited in the Gene Expression Omnibus database (GEO Series accession number GSE230291).

### RNA-seq data analysis

Raw reads from the sequencer were preprocessed to remove low-quality and adapter sequences prior to analysis, and reads for *Homo sapiens* (hg38) were processed using HISAT v2.1.0 [33]. HISAT uses two types of indexes for alignment: global whole genome indexes and tens of thousands of small local indexes. These two types of indexes are constructed using the same Burrows-Wheeler transform (BWT) and a graph FM index (GFM) as Bowtie2. Because these efficient data structures and algorithms are used, HISAT generates junctional alignments several times faster than Bowtie2 and BWT. The reference genome sequence and annotation data of *Homo sapiens* (hg38) were downloaded from the UCSC Table Browser (http://genome.uscs.edu). After sorting, StringTie v2.1.3b [34, 35] was used to assemble the sorted reads into transcripts and estimate their abundances. The results obtained provided an estimate of relative abundances as read counts of the expressed transcripts and genes in each sample.

Statistical analysis was conducted to identify DEGs using the gene abundance estimates. Genes with read count values ​​greater than zero in the sample were excluded. Log_2_ transformation was accomplished by adding 1 to each Read Count value of filtered genes, and log_2_ values were subjected to RLE normalization. The statistical significances of differential expression data were determined using nbinomWaldTest with DESeq2. The null hypothesis was that group fold changes were no different. False discovery rate (FDR) was controlled by adjusting p-values by applying the Benjamini-Hochberg algorithm. For the DEGs set, hierarchical clustering analysis was performed using perfect connectivity and Euclidean distance as measures of similarity.

### GO and KEGG pathway enrichment analyses

Gene ontology (GO) enrichment analysis (http://geneontology.org/) [36, 37] and Kyoto encyclopedia of genes and genomes (KEGG) pathway enrichment analysis (https://www.genome.jp/kegg/) [38–40] for a list of essential genes were performed based on the database for annotation, visualization, and integrated discovery (https://david.ncifcrf.gov/) [41, 42]. R (version 4.2.2) was used to visualize GO and KEGG pathway enrichment analyses of common DEGs. These two analyses provide integrated functional genomic annotations.

### Bioinformatic database analyses

The OncoLnc database (http://www.oncolnc.org/) contains survival data for 8,647 patients extracted from 21 cancer studies performed by the cancer genome atlas (TCGA), along with RNA-seq expression data for mRNAs and miRNAs from TCGA and lncRNA expression data from MiTranscriptome beta [43]. The prognostic value of OncoLnc expression data was investigated to determine the relationship between gene expression and NSCLC patient survival rates. Kaplan-Meier survival curves were constructed using OncoLnc to assess the impact of gene expression on prognosis [44].

The TNMplot database (https://www.tnmplot.com/) is a web platform that uses available transcriptome-level data sets to build an integrated database and enables the mining of this database by real-time comparisons of normal, tumor, and metastasis data for all genes [45]. The TNMplot database was used to compare gene expressions in normal and tumor tissues of NSCLC patients [46].

## Results

### Dysregulation of MALAT1 in NSCLC cell lines

**Fig. 1:**
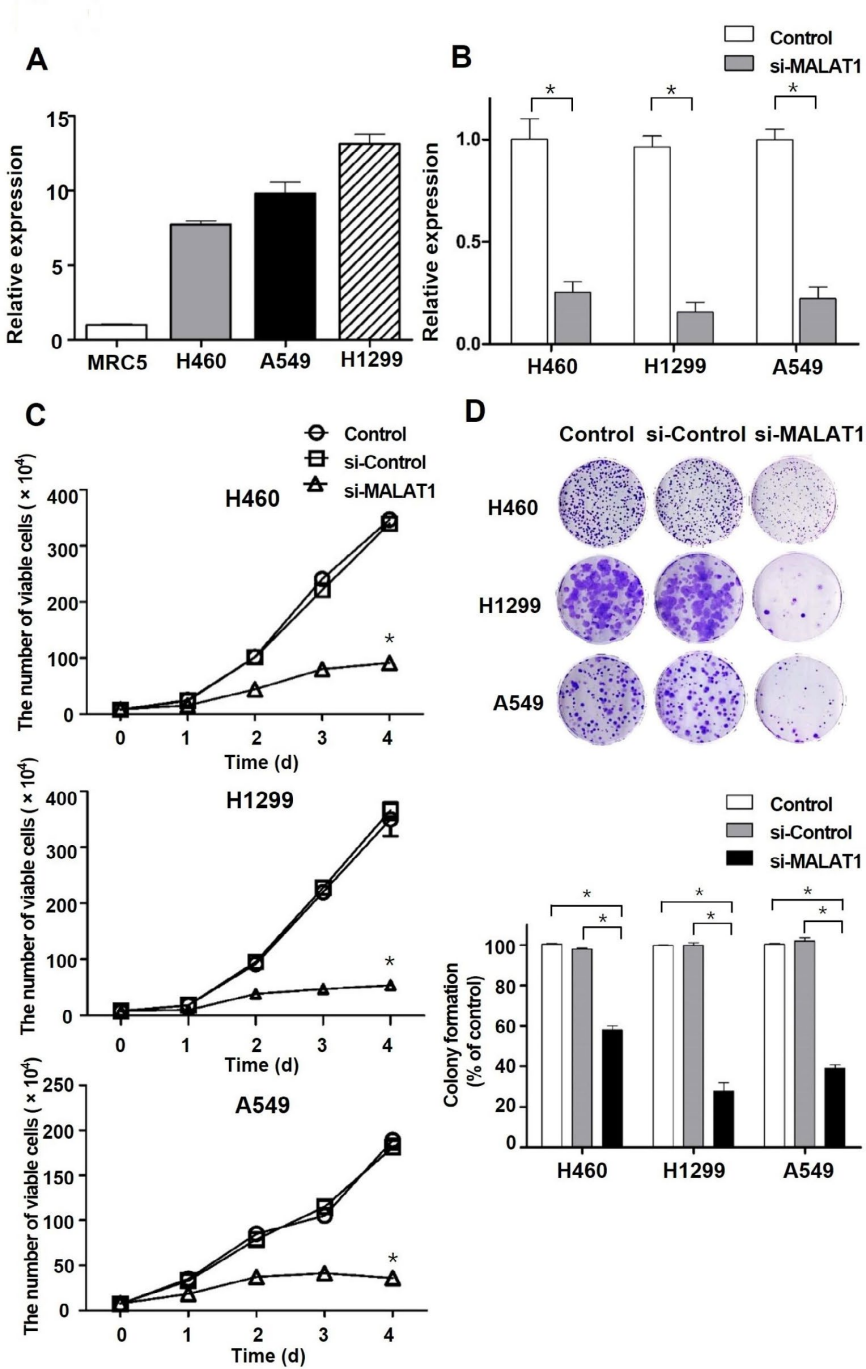
The effects of MALAT1 on relative expression level and cell viability in NSCLC cell lines. (**A**) Relative expression levels of MALAT1 in normal lung cell line (MRC5) and NSCLC cell lines (H460, A549, H1299) were quantified by RT-qPCR. (**B**) Relative expression levels of MALAT1 in the control groups and si-MALAT1 treatment groups were quantified by RT-qPCR. (**C**) Short-term effects of MALAT1 knockdown on NSCLC cell viability were determined by a cell counting assay. The number of viable cells was compared with control groups. (**D**) Long-term effects of MALAT1 knockdown on NSCLC cell viability were determined by a colony formation assay. Quantitative analyses of the number of NSCLC cell clones were performed with Image J. *p < 0.05

To identify MALAT1 expression status, we evaluated the expression levels of MALAT1 in a normal lung cell line, MRC5, and three NSCLC cell lines including H460, H1299, and A549. RT-qPCR showed the expression levels of MALAT1 were elevated in the three NSCLC cell lines versus MRC5 cells (Fig. 1A). In addition, RT-qPCR showed that the expression level of MALAT1 was significantly reduced in three NSCLC cell lines after si-MALAT1 treatment (Fig. 1B). Cell counting and colony formation assays were performed to verify that MALAT1 expression levels were related to viability. Cell counting assays were performed to assess the short-term effects of MALAT1 knockdown on NSCLC cell growth. Cell counts of untreated NSCLC controls significantly increased over time, whereas cell counts remained unchanged after MALAT1 knockdown (Fig. 1C). Colony formation assays were also performed to assess the long-term effects of MALAT1 downregulation on NSCLC cell proliferation, and MALAT1 knockdown was found to significantly reduce colony-forming ability (Fig. 1D). These results showed MALAT1 dysregulation in NSCLC cells responsible for NSCLC cell survival and proliferation.

### Inhibitory effects of MALAT1 knockdown on NSCLC cell motility

**Fig. 2:**
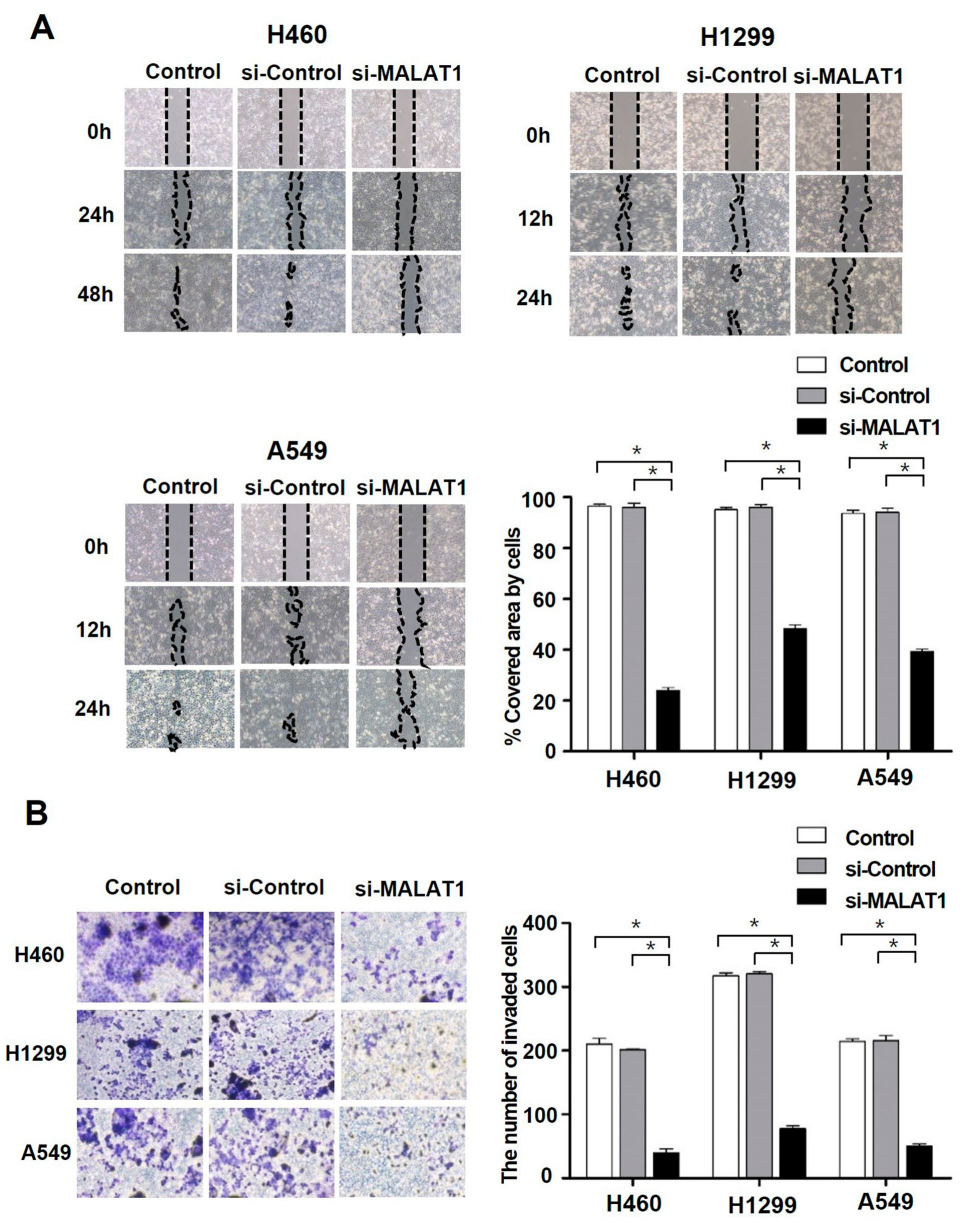
The effects of MALAT1 on cell motility in NSCLC cell lines. (**A**) The effects of MALAT1 knockdown on NSCLC cell migration were assessed by a wound healing assay. The graph shows the percentage of covered areas in si-MALAT1 groups compared to control groups. (**B**) The effects of MALAT1 knockdown on NSCLC cell invasion were assessed by a Transwell invasion assay. The number of invaded cells was compared with control groups. *p < 0.05

Wound healing and Transwell invasion assays were performed to assess the effects of MALAT1 on cell motility, a characteristic of cancer cells. Wound healing assays showed that migration ability was significantly suppressed in the si-MALAT1 group versus untreated controls (Fig. 2A). Furthermore, Transwell invasion assays showed that si-MALAT1 treatment significantly reduced NSCLC cell invasion versus untreated controls (Fig. 2B). These results suggest that MALAT1 facilitates metastasis in NSCLC.

### Profiling of DEGs in MALAT1 knockdown NSCLC cell lines

**Fig. 3:**
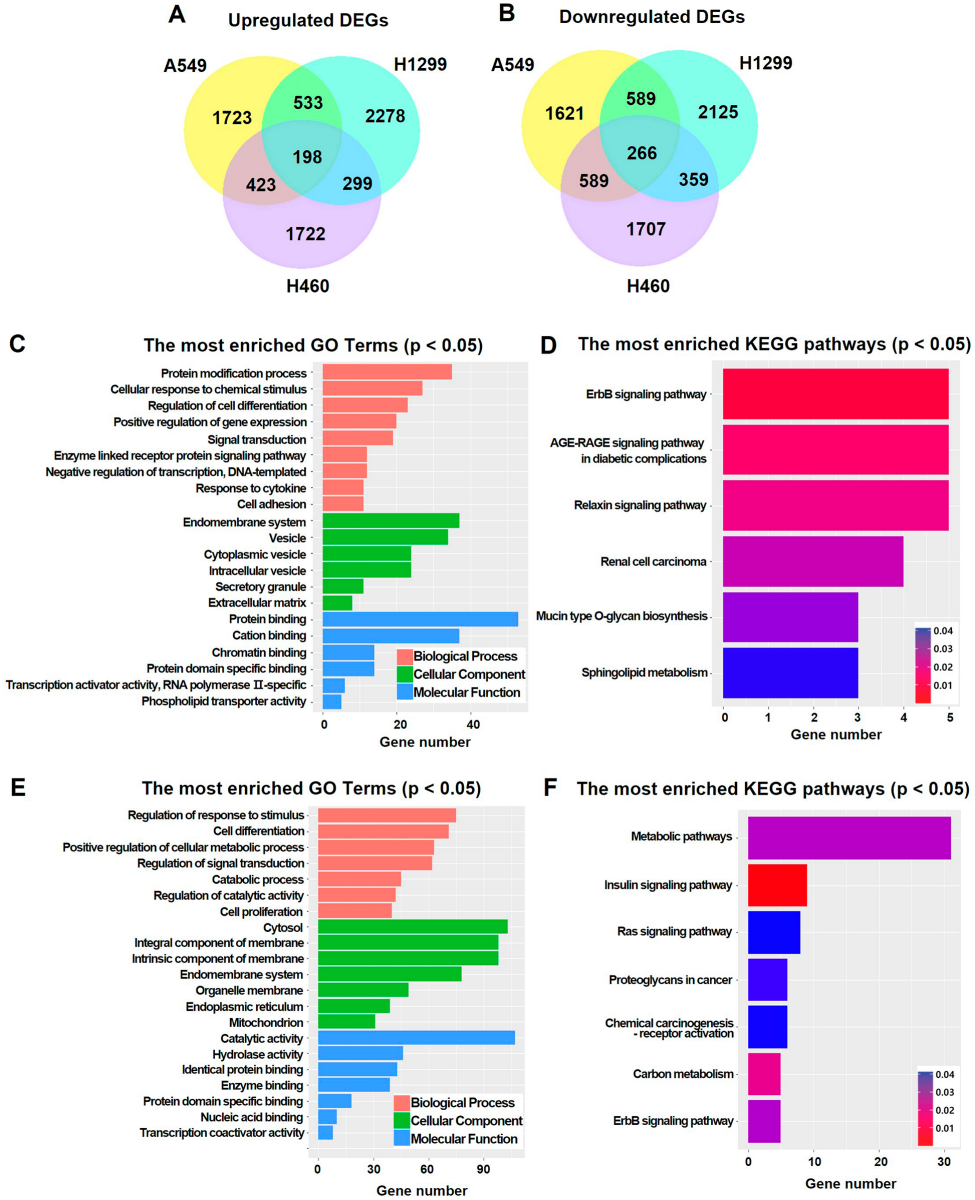
DEGs, GO enrichment, and KEGG pathway enrichment analyses in NSCLC cell lines after MALAT1 knockdown. (**A**) Venn Diagram represents the overlapped upregulated DEGs (log_2_ fold change ≥ 1.5). (**B**) Venn Diagram represents the overlapped downregulated DEGs (log_2_ fold change ≤ -1.5). (**C**) GO enrichment analysis of the upregulated DEGs shows the abundant terms based on p < 0.05 in the top ranking. (**D**) KEGG pathway enrichment analysis is shown for the upregulated DEGs. The color gradient from blue to red indicates p-value. (**E**) GO enrichment analysis of the downregulated DEGs shows the abundant terms based on p < 0.05 in the top ranking. (**F**) KEGG pathway enrichment analysis is shown for the downregulated DEGs. The color gradient from blue to red indicates p-value

RNA-seq analysis was performed to identify gene expression changes in the NSCLC cell lines after MALAT1 knockdown to identify genes associated with MALAT1. Only genes exhibiting expressional changes of 1.5-fold or more differences after MALAT1 knockdown (|log_2_ fold change| ≥ 1.5) were selected for further investigation (Supplementary Table 2). Common DEGs, that is, genes showing identical fold changes in the three NSCLC cell lines, were considered MALAT1-associated genes. Venn Diagram was used to screen common DEGs in the three NSCLC cell lines. MALAT1 knockdown resulted in the up- and downregulation of 198 and 266 common DEGs, respectively (Fig. 3A and B), which were considered putative tumor suppressor genes and oncogenes, respectively.

### Functional analyses of common DEGs

GO and KEGG pathway enrichment analyses were performed on the 198 upregulated and 266 downregulated common DEGs. The analyses showed that these DEGs are involved in tumor development. GO enrichment analysis showed upregulated DEGs were highly related to: ‘protein modification process’ (35 genes, p = 7.52e-03) and ‘cellular response to chemical stimulus’ (27 genes, p = 2.63e-02) of the Biological Process, ‘endomembrane system’ (37 genes, p = 4.02e-02) and ‘vesicle’ (34 genes, p = 2.68e-02) of the Cellular Component, and ‘protein binding’ (53 genes, p = 3.29e-02) and ‘cation binding’ (37 genes, p = 4.53e-02) of the Molecular Function (Fig. 3C). Protein modification processes play an important role in the regulation of cancer hallmarks [47]. KEGG pathway enrichment analysis showed that the upregulated common DEGs were highly associated with pathways including ‘ErbB signaling pathway’ (5 genes, p = 8.35e-03) and ‘AGE-RAGE signaling pathway in diabetic complications’ (5 genes, p = 1.45e-02) (Fig. 3D). The ErbB signaling pathway plays crucial roles in the development and progression of cancer [48].

GO enrichment analysis also showed that downregulated DEGs were highly related to: ‘regulation of response to stimulus’ (75 genes, p = 9.03e-04) and ‘cell differentiation’ (71 genes, p = 5.46e-03) of the Biological Process, ‘cytosol’ (103 genes, p = 1.31E-06) and ‘integral component of membrane’ (98 genes, p = 1.10e-03) of the Cellular Component, and ‘catalytic activity’ (107 genes, p = 8.40e-05) and ‘hydrolase activity’ (46 genes, p = 4.12e-02) of the Molecular Function (Fig. 3E). KEGG pathway enrichment analysis showed that downregulated common DEGs in NSCLC cell lines were highly related to pathways including ‘metabolic pathways’ (31 genes, p = 3.43e-02) (Fig. 3F). In addition, ‘Ras signaling pathway’ (8 genes, p = 4.80e-02) and ‘chemical carcinogenesis - receptor activation’ (6 genes, p = 4.51e-02) are signaling pathways directly related to cancer. Cell differentiation is one of the main characteristics of cancer cells [49], and metabolic pathways are known to be involved in their growth and survival [50, 51]. In addition, metabolic pathways are closely related to metabolism reprogramming, including the Warburg effect in cancer cells [52].

The DEGs included in the mentioned KEGG pathways have not been actively studied in NSCLC. However, each KEGG pathway has been reported to be closely related to cancer development, indicating the need for further research on MALAT1. Summarizing, GO enrichment and KEGG pathway enrichment analyses indicated that the identified DEGs are involved in tumorigenesis in NSCLC and that MALAT1 has an oncogenic function.

### MALAT1-induced DEGs associated with tumorigenesis

**Fig. 4:**
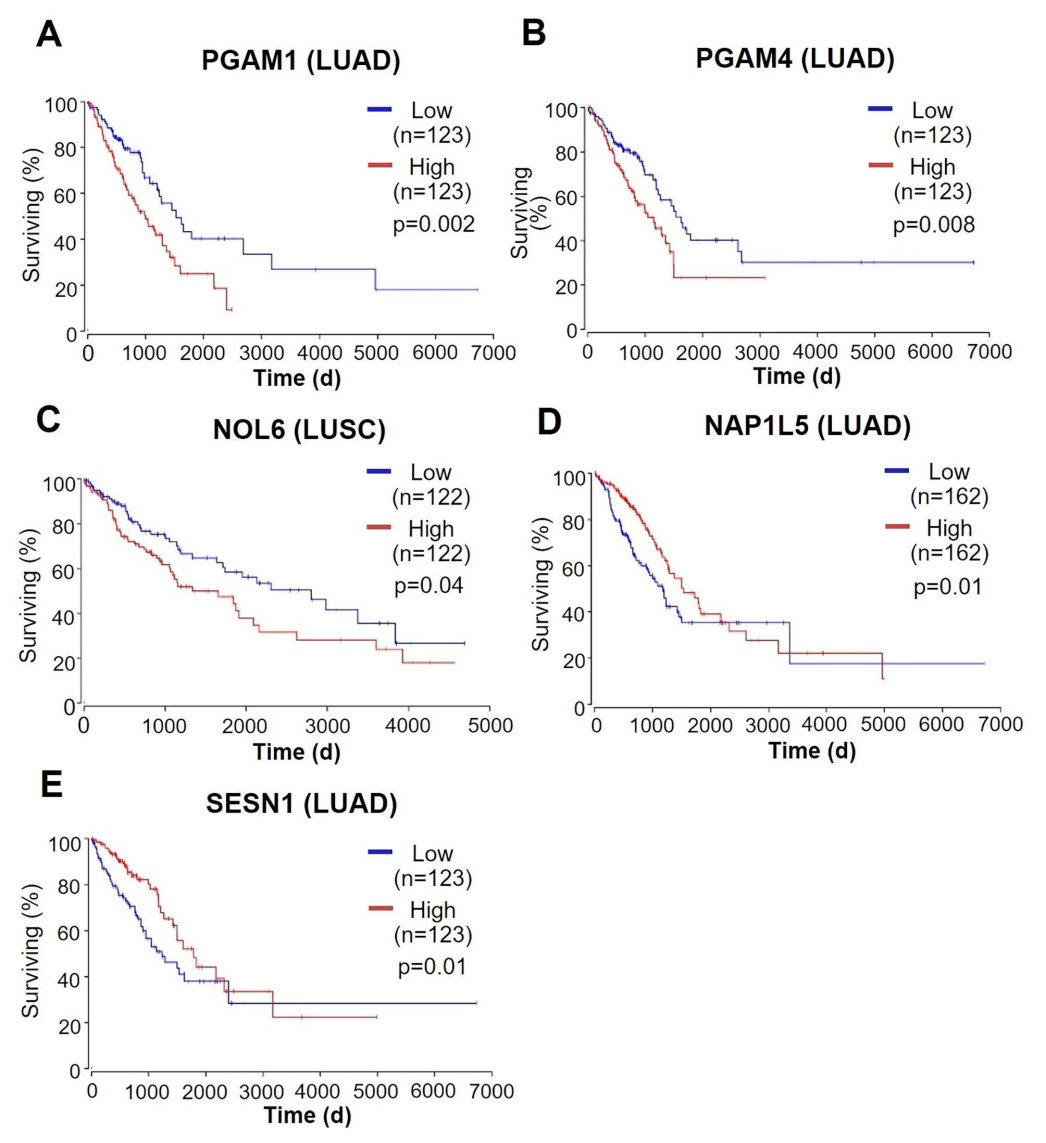
The results of survival analysis of five selected DEGs. (**A**) *PGAM1* (p = 0.002), (**B**) *PGAM4* (p = 0.008), (**C**) *NOL6* (p = 0.04), (**D**) *NAP1L5* (p = 0.01), and (**E**) *SESN1* (p = 0.01). Survival analysis in NSCLC patients was performed using the OncoLnc database

**Fig. 5:**
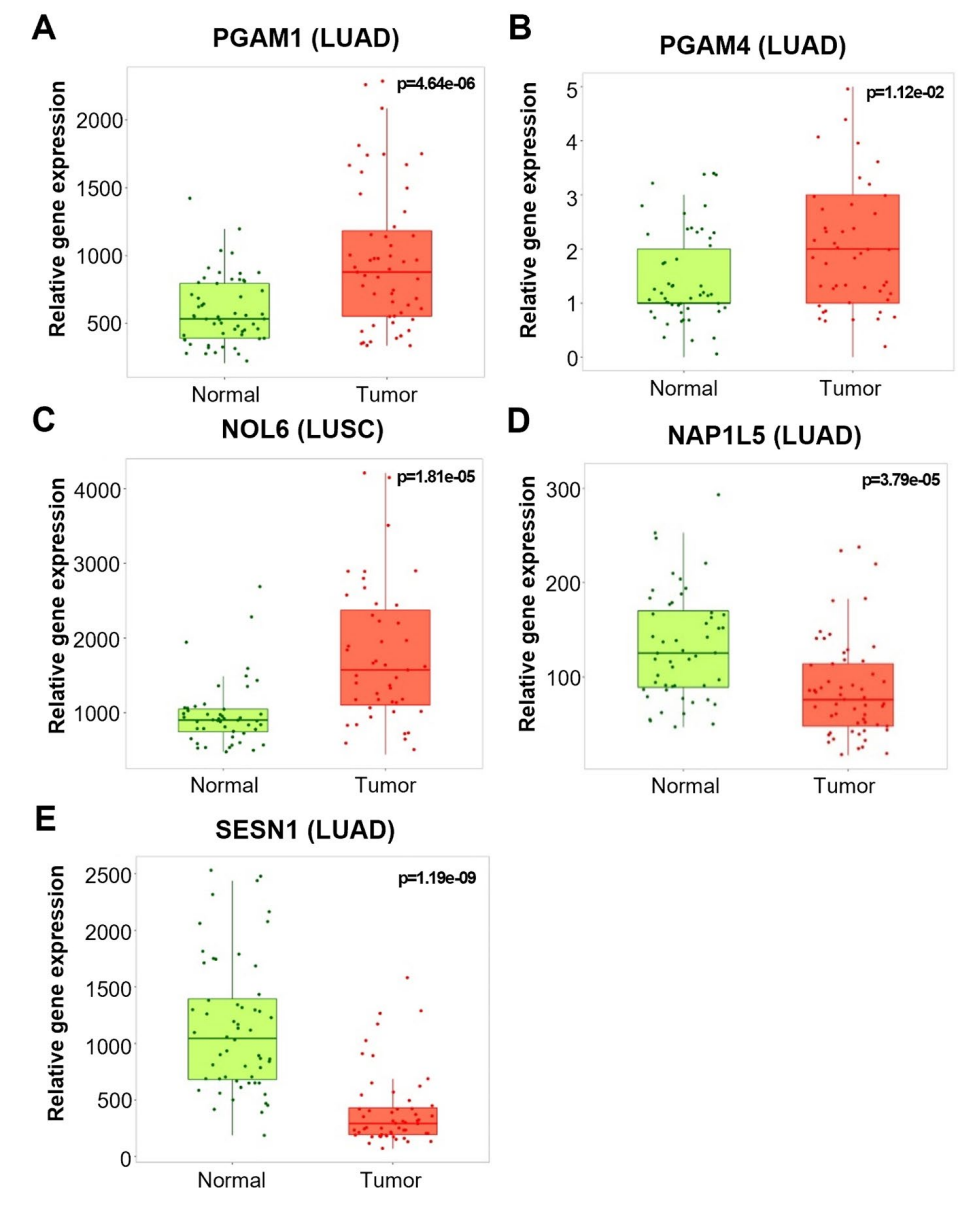
The results of gene expression analysis of five selected DEGs. (**A**) *PGAM1* (p = 4.64e-06), (**B**) *PGAM4* (p = 1.12e-02), (**C**) *NOL6* (p = 1.81e-05), (**D**) *NAP1L5* (p = 3.79e-05), and (**E**) *SESN1* (p = 1.19e-09). Gene expression analysis between NSCLC tissues and adjacent normal tissues was analyzed using the TNMplot database

We focused on ‘Molecular Function’ in GO terms to select genes predicted to be involved in tumorigenesis by interacting with MALAT1. Subsequently, the keywords for detailed screening were employed ‘metabolism’, ‘cell proliferation’ or ‘cell death’. As a result, five DEGs, phosphoglycerate mutase 1 (*PGAM1*), phosphoglycerate mutase 4 (*PGAM4*), nucleolar protein 6 (*NOL6*), nucleosome assembly protein 1 like 5 (*NAP1L5*), and sestrin1 (*SESN1*), were selected. For bioinformatic studies, survival analysis was performed using the OncoLnc database (http://www.oncolnc.org/) for selected DEGs on NSCLC patients with clinical impact (Fig. 4). We found that high expression levels of *PGAM1* and *PGAM4* in lung adenocarcinoma (LUAD) patients and *NOL6* in lung squamous cell carcinoma (LUSC) patients were associated with low overall survival rates. On the other hand, high expression levels of *NAP1L5* and *SESN1* increased overall survival in LUAD patients. In addition, we used the TNMplot database (https://tnmplot.com/) to analyze gene expression levels between tumors and adjacent tissues for the five DEGs (Fig. 5). Results confirmed that the expressional levels of *PGAM1* and *PGAM4* were elevated in the LUAD tissue group and that those of *NOL6* were increased in LUSC tissue group. On the other hand, the expressional levels of *NAP1L5* and *SESN1* were diminished in the LUAD tissue group. Thus, we identified three downregulated and two upregulated DEGs in the MALAT1 knockdown group, and bioinformatic analyses suggested these selected DEGs might be molecular biomarkers (Supplementary Table 3).

## Discussion

LncRNA MALAT1 is known to play a role in the tumorigenesis of NSCLC in previous studies [53]. To determine the oncogenic function of MALAT1 in lung cancer and identify genes interacting with MALAT1, we performed cell-based assays, transcriptome analyses, and bioinformatic analyses. First, H460, H1299, and A549 cell lines were used to confirm that MALAT1 acts as an oncogene in NSCLC, since the three cell lines have been previously reported to overexpress MALAT1 [54, 55]. By evaluating cell viability and motility, this study demonstrates that MALAT1 contributes to tumorigenesis in NSCLC. Furthermore, the study confirms that the expression level of MALAT1 is significantly higher in NSCLC cells than in normal lung cells. Cell counting and colony formation assays showed significant reductions in cell viability in MALAT1-knocked down NSCLC cells, and wound healing and Transwell invasion assays confirmed MALAT1 knockdown significantly suppressed cell motility. RNA-seq analysis showed that 198 common DEGs were upregulated by MALAT1 knockdown (putative tumor suppressors), and 266 common DEGs were downregulated (putative oncogenes). GO and KEGG pathway enrichment analyses showed that DEGs were involved in tumorigenesis.

GO enrichment analysis showed that the 198 common DEGs have tumor suppressor functions; GO terms included ‘protein modification process’, ‘cellular response to chemical stimulus’, ‘vesicle’, and ‘protein binding’ (Fig. 3C). The GO term ‘protein modification process’ includes histone deacetylase 9, a known tumor suppressor, the downregulation of which has been reported to promote disease progression, particularly in lung adenocarcinoma [56]. The GO term ‘cellular response to chemical stimulus’ includes the thyroid hormone receptor beta (*THRB*) gene, which has been observed in lung, skin, breast, head and neck, renal, cervical, ovarian, and testicular cancer. In lung cancer, it has been reported that THRB is genetically inactivated due to abnormal methylation [57]. The GO term ‘vesicle’ includes alkaline phosphatase (*ALPL*) and c-Myc. When ERK is inactivated by ALPL-induced dephosphorylation, the c-Myc degradation pathway is suppressed, and RhoA expression is consequently increased to promote metastasis. Therefore, increased ALPL would be expected to inhibit metastasis in LUAD [58]. The GO term ‘protein binding’ includes ATPase phospholipid transporting 8B1 (*ATP8B1*), and the downregulation of ATP8B1 was reported to promote the proliferation of LUSC cells and to inhibit apoptosis and aggravate the invasion and migration of these cells in vitro and in vivo [59]. In addition, KEGG pathway enrichment analysis showed that MALAT1-affected DEGs are related to ‘ErbB signaling pathway’, ‘sphingolipid metabolism’, and ‘Relaxin signaling pathway’ (Fig. 3D). The ErbB signaling pathways connect the binding of extracellular growth factor ligands to intracellular signaling pathways that regulate a variety of biological responses, including proliferation, differentiation, cell motility, and survival. In addition, evidence indicates that the ErbB receptor family and its downstream pathways modulate extracellular matrix components to regulate epithelial-mesenchymal transition, migration, and tumor invasion [60–62].

GO enrichment analysis showed 266 common DEGs with GO terms such as ‘regulation of response to stimulus’, ‘cell differentiation’, ‘cytosol’, and ‘catalytic activity’ have putative tumorigenic functions (Fig. 3E). The GO term ‘regulation of response to stimulus’ includes EPH receptor A2 (*EPHA2*), and the downregulation of EPHA2 reportedly reduces the proliferation and inhibits the migration of NSCLC cells [63]. The GO term ‘cell differentiation’ includes TBL1X/Y related 1 (*TBL1XR1*), and in NSCLC cells, TBL1XR1 has been shown to promote cell survival, proliferation, and metastasis [64]. The GO term ‘cytosol’ includes Keratin 80, which is known to promote the migration and invasion of NSCLC cells by regulating the TGF-β/SMAD pathway [65]. The GO term ‘catalytic activity’ includes C-C motif chemokine ligand 5, which activates αvβ3 integrin to promote migration as facilitated by the PI3K/Akt pathway through the activations of IKKα/β and NF-κB pathways in NSCLC cells [66]. KEGG pathway enrichment analysis identified ‘metabolic pathways’ as the most relevant (Fig. 3F). ‘Metabolic pathways’ include UDP-glucose 6-dehydrogenase (*UGDH*), tripartite motif containing 44 (*TRIM44*), and branched-chain amino acid transaminase 1 (*BCAT1*), and these genes are known to affect cancer development. UGDH promotes tumor metastasis by increasing the stability of snail family transcriptional repressor 1 mRNA [67]. TRIM44 reportedly increases proliferation and metastasis in NSCLC via the mTOR signaling pathway [68], and BCAT1 overexpression is known to activate proliferation, invasion, and Wnt signaling in NSCLC [69]. Thus, the study shows that DEGs and pathway changes caused by alterations in MALAT1 expression might promote or suppress NSCLC.

Although GO and KEGG pathway enrichment analyses predict that numerous genes regulated by MALAT1 may contribute to tumorigenesis, we selected five DEGs focusing on their molecular functions. Survival analysis and tissue gene expression analysis based on bioinformatic databases showed *PGAM1*, *PGAM4*, *NOL6*, *NAP1L5*, and *SESN1* had significant clinical impacts (Figs. 4 and 5). *PGAM1*, *PGAM4*, and *NOL6* have been reported to be tumorigenic in several cancers (Supplementary Table 3). PGAM1 is an oncogene that activates the TGF-β signaling pathway in NSCLC to increase cell proliferation and invasion [70], while being essential for energy metabolism and participating in carbohydrate transport, catalytic activity, growth, and development [71]. Although *PGAM4* and *NOL6* have not been actively studied in lung cancer, they are known to promote cell proliferation in other cancers. *PGAM4* was originally identified as a pseudogene derived from *PGAM1*, with which it retains 97.6% sequence identity [72]. PGAM4 exhibits putative amino acid identity with PGAM1 and contains the LxRHGExxxN motif for extensive PGAM enzymatic activity [73]. Despite a lack of study in lung cancer, PGAM4 is expected to promote tumor growth, such as in glioma [74]. NOL6 regulates cell proliferation and apoptosis by regulating the expressions of tumor protein p53 inducible protein 3, CDK4, and MCM7 in gastric cancer [75]. Furthermore, NOL6 is overexpressed in endometrial cancer and regulates TWIST1 expression, promotes migration, and reduces apoptosis [76]. In the present study, the expression levels of *PGAM1*, *PGAM4*, and *NOL6* were positively correlated with MALAT1 expression, and thus these genes probably interact with MALAT1 to promote NSCLC tumorigenesis. *NAP1L5* and *SESN1* have been shown to act as tumor suppressors in previous studies on cancer (Supplementary Table 3). NAP1L5 inhibits tumor growth and metastasis by inhibiting the PI3K/Akt/mTOR signaling pathway and myosin heavy chain 9 in hepatocellular carcinoma [77]. On the other hand, SESN1 is regulated by the tumor suppressor protein p53. In human lung adenocarcinoma cells, the inactivation of SESN1 supports cell proliferation and suppresses glucose starvation [78]. Since the expression levels of *NAP1L5* and *SESN1* are negatively correlated with MALAT1 expression, these genes are believed to interact with MALAT1 to inhibit tumorigenesis in NSCLC.

This study was revealed that MALAT1 is involved in the development of NSCLC by regulating the expressions of oncogenes and tumor suppressor genes. Thus, the usage of antisense oligonucleotides or siRNA to target MALAT1 might be an effective therapeutic strategy. In addition, GO and KEGG pathway enrichment analyses revealed that 464 DEGs were involved in cancer development, and 5 DEGs were selected as potential molecular biomarkers according to bioinformatic database analyses. However, further experimental evidence is needed; for example, the roles of *PGAM4* and *NOL6* have not been studied in lung cancer. Nevertheless, this study can give a better understanding of how MALAT1 acts in NSCLC and contribute to the identification of cancer-associated signaling pathways regulated by MALAT1. Moreover, it suggests that targeting MALAT1 could be a novel therapeutic strategy to inhibit tumorigenesis.

### Electronic supplementary material

Below is the link to the electronic supplementary material.

Supplementary Material 1

Supplementary Material 2

Supplementary Material 3

## Data Availability

The datasets presented in this study can be found in online repositories. The names of the repository/repositories and accession number(s) can be found below: https://www.ncbi.nlm.nih.gov/geo/, GSE230291.
