# Supplementary material for: MALAT1-regulated gene expression profiling in lung cancer cell lines
Source: BMC Cancer. 2023 Sep 4;23:818. doi: 10.1186/s12885-023-11347-7 (PMC10476395; doi:10.1186/s12885-023-11347-7)
Supplement: Supplementary file 1 — Supplementary Material 1 [file 12885_2023_11347_MOESM1_ESM.docx]

**Supplementary Table 1. Primers used in RT-qPCR for identifying MALAT1 expression levels**

| **Gene** | **Sequences (5′-3′)** | |
| --- | --- | --- |
|  | **Forward primer** | **Reverse primer** |
| U6 | CTCGCTTCGGCAGCACATA | CGAATTTGCGTGTCATCCT |
| MALAT1 | TGATAGCCAAATTGAGACAA | TTCAGGGTGAGGAAGTAAAA |
