# Supplementary material for: MALAT1-regulated gene expression profiling in lung cancer cell lines
Source: BMC Cancer. 2023 Sep 4;23:818. doi: 10.1186/s12885-023-11347-7 (PMC10476395; doi:10.1186/s12885-023-11347-7)
Supplement: Supplementary file 2 — Supplementary Material 2 [file 12885_2023_11347_MOESM2_ESM.docx]

**Supplementary Table 2. DEGs regulated by MALAT1 knockdown in NSCLC cell lines**

| **NSCLC**  **Cell line** | **DEGs after MALAT1 knockdown** | |
| --- | --- | --- |
|  | **Up-Regulation**  **(log_2_ fold change (FC) ≥ 1.5)** | **Down-Regulation**  **(log_2_ fold change (FC) ≤ -1.5)** |
| H460 | 2642 | 2921 |
| H1299 | 3308 | 3339 |
| A549 | 2877 | 3065 |
