# Supplementary material for: MALAT1-regulated gene expression profiling in lung cancer cell lines
Source: BMC Cancer. 2023 Sep 4;23:818. doi: 10.1186/s12885-023-11347-7 (PMC10476395; doi:10.1186/s12885-023-11347-7)
Supplement: Supplementary file 3 — Supplementary Material 3 [file 12885_2023_11347_MOESM3_ESM.docx]

**Supplementary Table 3. Regulatory level of DEGs expected to be involved in tumorigenesis**

| **Gene symbol** | **Regulated by**  **MALAT1** | **Expected function** | **Effect functions in various tumors** | **Reference** |
| --- | --- | --- | --- | --- |
| *PGAM1* | Down | Oncogenic | Promoting cell proliferation and invasion by activating TGF-β signaling in non-small cell lung cancer | [70] |
| *PGAM4* | Down | Oncogenic | Promoted cell viability, proliferation, and glycolysis through EP300-mediated modifications of H3K27ac in glioma | [74] |
| *NOL6* | Down | Oncogenic | Regulating cell proliferation and apoptosis by regulating the expression of TP53I3, CDK4 and MCM7 in gastric cancer | [75] |
| *NAP1L5* | Up | Tumor suppressive | Suppressing tumor growth and metastasis by inhibiting the PI3K/Akt/mTOR signaling pathway by regulating MYH9 in hepatocellular carcinoma | [77] |
| *SESN1* | Up | Tumor suppressive | Regulating cell proliferation and apoptosis by p53 in lung carcinogenesis | [78] |
